# Challenges of declarative modeling of conductance-based neurons in diverse simulation environments

**DOI:** 10.1186/1471-2202-14-S1-P94

**Published:** 2013-07-08

**Authors:** Ivan Raikov, Thomas G Close, Shyam Kumar S, Erik De Schutter

**Affiliations:** 1 Computational Neuroscience Unit, Okinawa Institute of Science and Technology, Okinawa, Japan; 2 University of Antwerp, Antwerp, Belgium

## 

There exist several declarative computer languages for describing computational neuroscience models. NeuroML[1] encompasses ion channels, cell morphology, and networks, NineML[2] is focused on hybrid spiking neurons, and SBML[3] has been successfully used to describe ion channel kinetics. However, despite the existence of these standards, only a handful of complex conductance-based neuronal models have been ported to a simulator-independent declarative formats, and even then they have been tested and used only in relatively similar cable equation solvers such as NEURON[4]. The present study is an attempt to answer the question whether several widely used models can be ported to a declarative language and used in diverse simulation environments such as NEST[5], a point neuron oriented simulator, or general-purpose numerical environments such as GNU Octave[6].

We present the results of our work on porting several complex conductance-based neuronal models to a prototype layer-oriented declarative model description language [7]. The types of cells included in the study are Purkinje cell [8], Golgi cell [9], cerebellar granule cell [10], CA1 and CA3 pyramidal cell [11,12]. The models chosen are among or derived from the 30 most frequently downloaded models from ModelDB [13](T Morse: Personal Communication). The study involves the simulation of current injection and voltage clamp under NEURON, NEST and Octave and quantifying the existing differences in voltage and current traces. In cases where the original model is multi-compartmental, only a single-compartment soma variant is considered.

In the study, we attempt to quantify some of the important differences that exist between simulators, and we present a code generation approach that can solve the challenges caused by these differences. Furthermore, we highlight practical issues encountered while developing a convenient Python wrapper class for model code generated from the prototype language. This work is a step towards establishing a significant body of declarative models of neurons and identifies some of the issues related to interoperability of diverse neuroscience software.
